# Vancomycin efficiency and safety of a dosage of 40–60 mg/kg/d and corresponding trough concentrations in children with Gram-positive bacterial sepsis

**DOI:** 10.3389/fcimb.2023.1117717

**Published:** 2023-04-03

**Authors:** Lengyue Peng, Ziyao Guo, Guangli Zhang, Xiaoyin Tian, Ruixue Gu, Qinyuan Li, Yuanyuan Li, Zhengxiu Luo

**Affiliations:** ^1^ Department of Respiratory Medicine Children’s Hospital of Chongqing Medical University, National Clinical Research Center for Child Health and Disorders, Ministry of Education Key Laboratory of Child Development and Disorders, Chongqing Key Laboratory of Pediatrics, Chongqing, China; ^2^ Department of Child Care, The First People’s Hospital of Chongqing Liangjiang New Area, Chongqing, China

**Keywords:** vancomycin, Gram-positive bacterial, children, tough concentrations, dosages

## Abstract

**Background:**

Optimal vancomycin trough concentrations and dosages remain controversial in sepsis children. We aim to investigate vancomycin treatment outcomes with a dosage of 40-60 mg/kg/d and corresponding trough concentrations in children with Gram-positive bacterial sepsis from a clinical perspective.

**Methods:**

Children diagnosed with Gram-positive bacterial sepsis and received intravenous vancomycin therapy between January 2017 and June 2020 were enrolled retrospectively. Patients were categorized as success and failure groups according to treatment outcomes. Laboratory, microbiological, and clinical data were collected. The risk factors for treatment failure were analyzed by logistic regression.

**Results:**

In total, 186 children were included, of whom 167 (89.8%) were enrolled in the success group and 19 (10.2%) in the failure group. The initial and mean vancomycin daily doses in failure group were significantly higher than those in success group [56.9 (IQR =42.1-60.0) *vs*. 40.5 (IQR =40.0-57.1), P=0.016; 57.0 (IQR =45.8-60.0) *vs*. 50.0 (IQR =40.0-57.6) mg/kg/d, P=0.012, respectively] and median vancomycin trough concentrations were similar between two groups [6.9 (4.0-12.1) *vs*.7.3 (4.5-10.6) mg/L, P=0.568)]. Moreover, there was no significant differences in treatment success rate between vancomycin trough concentrations ≤15 mg/L and >15 mg/L (91.2% *vs*. 75.0%, P=0.064). No vancomycin-related nephrotoxicity adverse effects occurred among all enrolled patients. Multivariate analysis revealed that a PRISM III score ≥10 (OR =15.011; 95% CI: 3.937-57.230; P<0.001) was the only independent clinical factor associated with increased incidence of treatment failure.

**Conclusions:**

Vancomycin dosages of 40-60 mg/kg/d are effective and have no vancomycin-related nephrotoxicity adverse effects in children with Gram-positive bacterial sepsis. Vancomycin trough concentrations >15 mg/L are not an essential target for these Gram-positive bacterial sepsis patients. PRISM III scores ≥10 may serve as an independent risk factor for vancomycin treatment failure in these patients.

## Introduction

Sepsis, a life-threatening infection due to a dysregulated host response to infections, is considered as one of the leading causes of morbidity, mortality and accounts for a heavy socioeconomic burden in children globally (Weiss et al., 2020). An estimate 48 children per 100 000 population suffer sepsis, 22 children per 100 000 population suffer severe sepsis, and 2202 neonates per 100 000 livebirths develop neonatal sepsis (Fleischmann-Struzek et al., 2018). Mortality ranged from 1% to 5% for sepsis and 9% to 20% for severe sepsis in children (Fleischmann-Struzek et al., 2018). A 20-year antimicrobial surveillance has shown that Gram-positive bacteria are the predominant cause of sepsis (Diekema et al., 2019). Undoubtedly, early identification and appropriate treatment are critically important for children with sepsis (Weiss et al., 2020). Rigorous evaluation of optimal antimicrobial and other therapeutic strategies are correlated with the clinical outcomes (Randolph et al., 2019).

Vancomycin, as a major glycopeptide antibiotic be used to treat severe infections caused by methicillin-resistant *Staphylococcus aureus* (MRSA) and methicillin-resistant *Staphylococcus epidermidis*, as well as penicillin-resistant *Corynebacterium jeikeium*, *Streptococcus pneumoniae*, and *Clostridium difficile*, is currently recommended for children with severe Gram-positive bacterial infections (Liu et al., 2011; Rubinstein and Keynan, 2014). Undisputedly, appropriate vancomycin dosages and trough concentrations are crucial for children with Gram-positive bacterial sepsis. The Infectious Diseases Society of America (IDSA) guidelines recommend children with serious or invasive infectious disease should be treated with vancomycin at a dosage of 15 mg/kg/d every 6 h (Liu et al., 2011). Vancomycin exhibits time-dependent bactericidal effect, meaning that its antibacterial activity dependent on the time that the concentration of the drug in the body is above the minimum inhibitory concentration (MIC). Thus area under the curve (AUC) over 24 hours to MIC ≥400 is the best index to evaluate clinical efficacy and ensure safety for children being treated with vancomycin (Rybak et al., 2020). However, measuring AUC/MIC by traditional method is not practical in clinical setting as it needing to obtain multiple serum vancomycin concentrations. Therefore, to attain the recommended target AUC/MIC ≥400, the IDSA guidelines recommended that targeting vancomycin trough concentration of 10-20 mg/L in pediatrics: 10-15 mg/L for uncomplicated infections and 15-20 mg/L for serious infections (Liu et al., 2011). However, the IDSA guidelines do acknowledge that limited data is available to evaluate the efficacy and safety of the aforementioned vancomycin dosages and trough concentrations for children (Liu et al., 2011).

Several studies have investigated the efficacy and safety of vancomycin trough concentrations of 10-20 mg/L (Frymoyer et al., 2013; Liang et al., 2018; Tkachuk et al., 2018; Peng et al., 2021). From a pharmacokinetic perspective, the trough concentration of 6-10 mg/L is likely sufficient to achieve AUC/MIC ≥400 in children (Frymoyer et al., 2013; Tkachuk et al., 2018). Another prospective multicenter study revealed that increasing vancomycin trough concentrations to 15-20 mg/L couldn’t benefit Chinese patients with complicated infections in children (Liang et al., 2018). Moreover, a population-based pharmacokinetic modeling study demonstrated that vancomycin trough concentrations ≥15 mg/L were independently associated with a above 2.5-fold increased risk of nephrotoxicity in children (Le et al., 2015). However, two previous studies reported that maintaining trough concentrations of 15-20 mg/L was not correlated with increased risk of nephrotoxicity in children (Cies and Shankar, 2013; Matson et al., 2015). About vancomycin dosages, a revised vancomycin consensus guideline recommended that the initial vancomycin dosage should be 60-80 mg/kg/day to achieve AUC/MIC ≥ 400 for children with normal renal function and suspected serious MRSA infections (Rybak et al., 2020). While Liang et al. (Liang et al., 2018) found an average vancomycin dosage of 37.7 mg/kg/d could treat 96% Gram-positive bacterial infectious children successfully.

As the optimal vancomycin trough concentrations and dosages remain controversial in children and most of the abovementioned studies discussed from a pharmacokinetic perspective (Frymoyer et al., 2013; Le et al., 2015; Tkachuk et al., 2018; Rybak et al., 2020), this retrospective cohort study aimed to explore the efficiency and safety of current vancomycin dosages and corresponding trough concentrations in children with Gram-positive bacterial sepsis from a clinical perspective.

## Methods

### Study site and study population

We performed a retrospective cohort study in the Children’s Hospital of Chongqing Medical University. The hospital is a 2480-bed tertiary teaching hospital in Chongqing, China and ranks among the top three domestic children’s hospitals (rank list: http://top100.imicams.ac.cn/home). We retrospectively enrolled 186 children hospitalized between January 2017 and June 2020. The inclusion criteria were all of the following: (i) aged 1m-18 years, (ii) diagnosed with sepsis or septic shock according to the 2020 Pediatric Surviving Sepsis Campaign guidelines (Weiss et al., 2020), (iii) at least one Gram-positive pathogen was obtained from sterile sites with accompanying clinical signs, (iv) received intravenous vancomycin administered as intermittent infusion for at least 48 hours, (v) serum trough concentrations were collected at steady state conditions. If multiple trough concentrations were available, only the first trough was included in this study. The exclusion criteria included any of the following: (i) children with incomplete clinical information, (ii) renal impairment at the initial administration of vancomycin, (iii) considered to have Gram-positive bacteria colonization or pollution, (iv) the vancomycin dosing regimen was not 40-60 mg/kg/d. (v) received linezolid or teicoplanin therapy ≥24 hours concurrently when undergoing vancomycin treatment. This study was approved by the institutional review board of the Children’s Hospital of Chongqing Medical University (Approved ID: 2022-463). A waiver of informed individual consent was granted given the retrospective design of this study.

### Data collection

Trained staff used a standardized data collection form to extract patients’ information from the electronic medical records. Patients' demographic characteristics (age, weight, gender), source of infection, comorbid illnesses, culture pathogens, the MIC values, baseline serum creatinine, liver and kidney function before and after vancomycin treatment, glucocorticoids and gamma globulin administration, laboratory data at the beginning of vancomycin treatment [C-reactive protein (CRP), procalcitonin (PCT), hemoglobin, neutrophil, albumin], the Pediatric Risk of Mortality (PRISM) III scores, concomitant antibiotics, antibiotics used before vancomycin therapy and vancomycin-related details (daily dose, duration and trough concentrations) were collected.

### Vancomycin administration and therapeutic drug monitoring

All enrolled children received a starting vancomycin (Vianex S.A., Athens, Greece) dosage of 40-60 mg/kg/day intravenously (maximum daily dose <2000 mg) as recommended by drug instructions and IDSA guidelines. Clinicians adjusted vancomycin dosage according to the therapeutic drug monitoring (TDM). IDSA recommends that the TDM target for the vancomycin trough concentration is 10-15 mg/L for common infections and 15-20 mg/L for children with complicated infections (Liu et al., 2011). However, clinicians could continue at the same vancomycin dose and track the concentration weekly if patients’ clinical and microbiological outcomes improved when trough concentrations were lower than the recommended range. Serum trough concentrations were obtained at steady state conditions [defined as any concentrations had been obtained within 1 hour after at least the third scheduled vancomycin administration (Heble et al., 2013)]. If multiple trough concentrations were available, only the first trough was included in this study. The chemiluminescence immunoassay (Abbott Laboratories, Chicago, IL, USA), which has an analytical range of 0.0–100.0 mg/L with a between-run coefficient of variation of <15% throughout the analytical range, was used to analyze vancomycin trough concentrations. A central laboratory was responsible for testing all serum samples within 24 hours, and intra- and inter-batch quality control was measured following the China National Accreditation Service for Conformity Assessment standard.

### Definition

The outcome of treatment success included clinical cure and microbiological cure. Clinical cure was defined as the improvement of patient’s presenting signs, symptoms and laboratory data (Ma et al., 2020). Microbiological cure was defined as the clearance of the original microbiological from the infection site during or up to 14 days after the administration of vancomycin therapy (Ma et al., 2020). Treatment failure included any of the following: (i) culture the original microorganism from the infection site (Liang et al., 2018), (ii) a lack of improvement of clinical symptoms, signs, and laboratory data requiring a change of a new antibiotic with a similar spectrum due to lack of response (Liang et al., 2018; Ma et al., 2020), (iii) readmission within 14 days of discharge for infection recurrence (Goldstein et al., 2013), (iv) all-cause mortality (Tong et al., 2020). When the children had renal impairment and occurred acute kidney injury (AKI) during vancomycin treatment, vancomycin-related nephrotoxicity was diagnosed. According to Kidney Disease Improving Global Guidelines (KDIGO) Clinical Practice Guidelines, AKI was defined as an increase in serum creatinine of ≥26.5 µmol/L within 48 hours or a known or presumed increase in serum creatinine to ≥1.5 times the baseline within 7 days, or a urine volume <0.5 mL/kg/hour for 6 hours (Khwaja, 2012). Baseline serum creatinine was defined as the lowest level obtained 1 week prior to the initial administration of vancomycin (Ma et al., 2020). Vancomycin-related nephrotoxicity was assessed after 48 hours of initiation of vancomycin to avoid confounding associated with increases in serum creatinine due to sepsis rather than exposure to vancomycin (Goldstein et al., 2013). The source of infection was identified by reviewing the clinical records, radiographic studies, surgical findings and laboratory records of the children (Yang et al., 2018). The severity of illness estimated by the PRISM III scores (Pollack et al., 1996) at the beginning of vancomycin treatment.

### Outcomes

The primary outcome was to investigate vancomycin efficiency and safety with current dosages and corresponding trough concentrations in children with Gram-positive bacterial sepsis. Secondary outcomes included the risk factors for treatment failure in this population.

### Statistical analysis

Descriptive statistics were performed to evaluate all variables of interest. The median [interquartile ranges (IQRs)] or mean [standard deviation (SD)] was used to describe the quantitative variables, whereas the categorical variables were displayed as counts (n) and percentages (%). In the univariate analysis, the chi-square or Fisher exact test was assessed for categorical variables, the Mann-Whitney *U* analysis was employed for non-normally distribution variables and two-sample t-test was used to analyses the normally distribution variables. Wilcoxon signed-rank test or paired sample t-test was used to examine any significant difference in the pre- and post-vancomycin treatment in kidney and liver function. Univariate and multivariable logistic regression analyses were performed to identify the independent association between potential factors and treatment failure, and odds ratios (ORs) and 95% confidence intervals (CIs) were calculated. We employed a receiver operating characteristic (ROC) curve and the maximum Youden’s index to select the appropriate cutoff value of PRISM III scores and we also evaluated the specificity and sensitivity of the prediction model. All statistical testing was performed using IBM SPSS Statistics 22 (SPSS Inc., Chicago, IL, USA). P values were from 2-sided analyses, and a P value <0.05 was deemed statistically significant.

## Results

### Enrollment and basic characteristics of all enrolled patients

**Figure 1:**
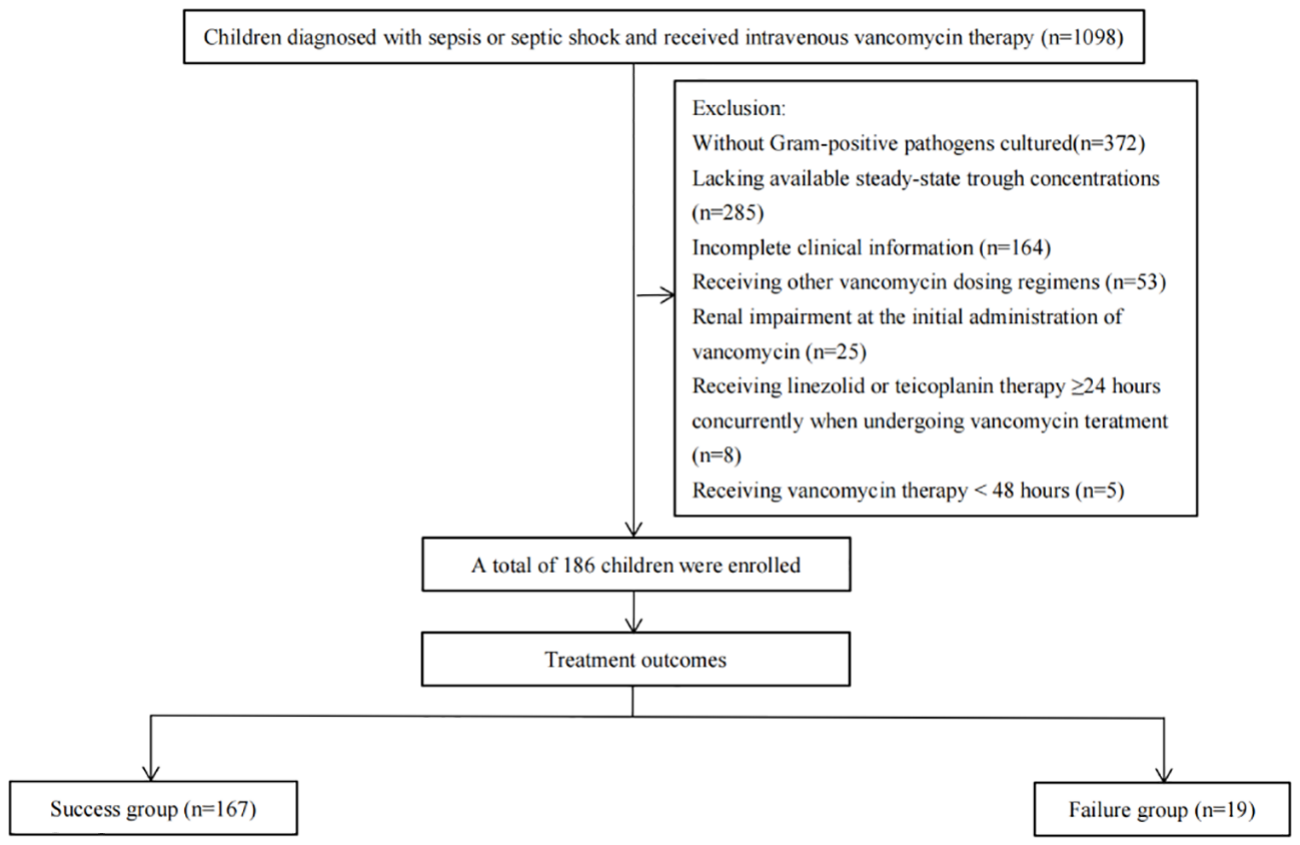
Flow chart of patients enrollment.

During this study, 1098 patients were screened for eligibility. Of these, 372 patients were excluded due to no Gram-positive pathogens cultured, 285 patients for lacking available steady-state trough concentrations, 164 patients for having incomplete clinical information, 53 patients for receiving other vancomycin dosing regimens, 25 patients for having renal impairment at the initial administration of vancomycin, 8 patients for receiving linezolid or teicoplanin therapy ≥24 hours concurrently when undergoing vancomycin treatment and 5 patients for receiving vancomycin therapy <48 hours. The remaining 186 patients were included with 167 (89.8%) in the success group, while the remaining 19 (10.2%) were in the failure group (**Figure 1**).

The median age of all patients was 2.0 (IQR =0.8-4.6) years and male gender accounted for 53.2% (99/186) of the patients. The initial and mean vancomycin daily dose was 41.7 (IQR =40.0-57.7) mg/kg/day and 50.0 (IQR =41.5-58.2) mg/kg/day, respectively. The median duration of vancomycin therapy was 12.0 (IQR =8.0-17.0) days. Bacteremia (56.5%) was the most common focus of infections. This was followed by respiratory system infections (27.4%) and skin and soft tissue infections (18.8%). The *Streptococcus pneumoniae* (21.0%) accounted for the most common infectious pathogen, followed by Methicillin susceptible *Staphylococcus aureus* (MSSA) (17.7%) and MRSA (14.0%). The treatment success rate was 89.8% (167/186). Moreover, no vancomycin-related nephrotoxicity adverse effects were observed in all enrolled patients (**Table 1**). All patients in the success group achieved microbial clearance, while in the failure group, 26.3% (5/19) patients cultured the original microorganism from the infection site which leaded to requiring antibiotic alteration or death and the remaining 73.7% (14/19) patients did not receive microbiological retesting.

**Table 1:** Basic characteristics and demographic data of all enrolled patients.

Variables	All patients (n = 186)
Demographics	
Male, n (%)	99 (53.2)
Age (years), median (IQR)	2.0 (0.8-4.6)
Body weight (kg), median (IQR)	11.8 (8.0-16.5)
Vancomycin therapy	
Initial vancomycin daily dose (mg/kg/day), median (IQR)	41.7 (40.0-57.7)
Mean vancomycin daily dose (mg/kg/day), median (IQR)	50.0 (41.5-58.2)
Duration of vancomycin therapy (days), median (IQR)	12.0 (8.0-17.0)
Trough concentration (mg/L), median (IQR)	7.3 (4.5-10.8)
PRISM III scores, median (IQR)	5.0 (3.0-9.0)
Infection sites, n (%)^a^	
Bacteremia	105 (56.5)
Respiratory system	51 (27.4)
Skin and soft tissue	35 (18.8)
Nervous system	25 (13.4)
Bone and joint	22 (11.8)
Other system	11 (5.9)
Microorganisms, n (%)	
* Streptococcus pneumoniae*	39 (21.0)
* MSSA*	33 (17.7)
* MRSA*	26 (14.0)
* Staphylococcus epidermidis*	23 (12.4)
* Staphylococcus hominis*	19 (10.2)
* Enterococcus faecium*	10 (5.4)
* Staphylococcus haemolyticus*	8 (4.3)
Other Gram-positive bacteria	28 (15.1)
Gram negative bacteria co-infections	32 (17.2)
Fungal co-infection	2 (1.1)
Underlying conditions, n (%)	
Hematologic malignancy	38 (20.4)
Congenital heart disease	16 (8.6)
Nephrotoxicity, n (%)	0 (0)
Treatment success, n (%)	167 (89.8)

a The total percentage may >100 due to a patient having multiple infectious sites.

IQR, interquartile range; PRISM, Pediatric Risk of Mortality; MSSA, Methicillin susceptible Staphylococcus aureus; MRSA, Methicillin-resistant Staphylococcus aureus.

### Basic characteristics of success and failure groups

**Table 2** presented the basic characteristics of success and failure groups. The success group patients had a significantly longer duration of vancomycin therapy when compared to that in the failure group [12.0 (IQR =9.0-17.0) *vs.* 8.0 (IQR =7.0-14.0) days, P=0.037]. Intriguingly, initial and mean vancomycin daily doses in the failure group were significantly higher than those in the success group [56.9 (IQR =42.1-60.0) *vs*. 40.5 (IQR=40.0-57.1), P=0.016; 57.0 (IQR =45.8-60.0) *vs*. 50.0 (IQR =40.0-57.6) mg/kg/d, P=0.012, respectively]. The albumin level was remarkably lower in treatment failure group patients when compared to those who were treated successfully [(29.9 ± 7.2) *vs*. (33.6 ± 7.0) g/dL, P=0.030]. The success group patients had a significantly lower proportion of septic shock than that in failure group (13.8% *vs.* 42.1%, P=0.005). Moreover, PRISM III scores in success group were significantly lower when compared to those in the failure group [5.0 (IQR =3.0-9.0) *vs*. 12.0 (IQR =5.0-23.0), P<0.001]. The failure group patients had a significantly higher proportion of Gram-negative bacteria co-infections than that in the success group (36.8% *vs.* 15.0%, P=0.017). Also, carbapenems were more commonly used concomitant with vancomycin therapy in failure group when compared to those in success group (68.4% *vs*. 43.1%, P=0.036). Demographics, source of infections, underlying conditions, other treatments, MIC values and antibacterial drugs before vancomycin therapy were similar between two groups (P>0.05). No patients had AKI and liver impairment in two group patients with a vancomycin dosage of 40-60 mg/kg/d and corresponding trough concentrations (**Table S1**). **Tables S2**, **S3** presented the data before and after concentration adjustment in the two group patients.

**Table 2:** Basic characteristics of success and failure groups.

Variables	Success group (n = 167)	Failure group (n = 19)	P Value
Demographics			
Age (years), median (IQR)	2.0 (0.9-4.6)	1.7 (0.8-11.1)	0.514
Male, n (%)	88 (52.7)	11 (57.9)	0.667
Body weight (kg), median (IQR)	12.0 (8.5-16.5)	9.5 (6.5-31.0)	0.298
Vancomycin therapy			
Trough concentration (mg/L), median (IQR)	7.3 (4.5-10.6)	6.9 (4.0-12.1)	0.568
Duration of vancomycin therapy (days), median (IQR)	12.0 (9.0-17.0)	8.0 (7.0-14.0)	0.037
Initial vancomycin daily dose (mg/kg/day), median (IQR)	40.5 (40.0-57.1)	56.9 (42.1-60.0)	0.016
Mean vancomycin daily dose (mg/kg/day), median (IQR)	50.0 (40.0-57.6)	57.0 (45.8-60.0)	0.012
Laboratory data			
Neutrophil (*10^9^/L), median (IQR)	5.8 (1.0-12.9)	10.9 (2.7-18.3)	0.070
CRP (mg/L), median (IQR)	40.0 (17.0-72.0)	69.0 (9.0-83.0)	0.214
PCT (ng/ml), median (IQR)	1.2 (0.3-6.2)	7.8 (0.7-47.0)	0.064
Hemoglobin, (g/dL), (SD)	95.8 (15.8)	93.8 (16.1)	0.619
Albumin (g/dL), (SD)	33.6 (7.0)	29.9 (7.2)	0.030
Infection site^a^, n (%)			
Bacteremia	94 (56.3)	11 (57.9)	0.893
Respiratory system	44 (26.3)	7 (36.8)	0.331
Skin and soft tissue	34 (20.4)	1 (5.3)	0.132
Nervous system	20 (12.0)	5 (26.3)	0.145
Bone and joint	19 (11.4)	3 (15.8)	0.476
Other system	11 (6.6)	0 (0)	0.607
Microorganisms, n (%)			
Streptococcus pneumoniae	35 (21.0)	4 (21.1)	1.000
MSSA	29 (17.4)	4 (21.1)	0.751
Staphylococcus epidermidis	22 (13.2)	1 (5.3)	0.476
MRSA	22 (13.2)	4 (21.1)	0.312
Staphylococcus hominis	18 (10.8)	1 (5.3)	0.698
Enterococcus faecium	10 (6.0)	0 (0)	0.602
Staphylococcus haemolyticus	7 (4.2)	1 (5.3)	0.585
Other Gram-positive bacteria	24 (14.4)	4 (21.1)	0.485
Gram negative bacteria co-infections	25 (15.0)	7 (36.8)	0.017
Fungal co-infections	2 (1.2)	0 (0)	1.000
Underlying conditions, n (%)			
Hematologic malignancy	37 (22.2)	1(5.3)	0.130
Congenital heart disease	14 (8.4)	2 (10.5)	0.670
Other treatments, n (%)			
Glucocorticoids	17 (10.2)	1 (5.3)	0.492
Gamma globulin	43 (25.7)	8 (42.1)	0.173
Severity of the disease			
Septic shock, n (%)	23 (13.8)	8 (42.1)	0.005
PRISM III scores, median (IQR)	5.0 (3.0-9.0)	12.0 (5.0-23.0)	<0.001
MIC ≤ 1, n (%)	154 (92.2)	18 (94.7)	1.000
Antibacterial drugs before vancomycin therapy^b^, n (%)			
Penicillins+β-lactamase inhibitors	60 (35.9)	3 (15.8)	0.123
Third generation Cephalosporins	56 (33.5)	10 (52.6)	0.099
Second generation Cephalosporins	25 (15.0)	2 (10.5)	1.000
Carbapenems	33 (19.8)	5 (26.3)	0.549
Fourth generation Cephalosporins	12 (7.2)	0 (0.0)	0.615
First generation Cephalosporins	9 (5.4)	0 (0)	0.601
Macrolides	6 (3.6)	2 (10.5)	0.191
Metronidazole	4 (2.4)	1 (5.3)	0.420
Penicillins	5 (3.0)	2 (10.5)	0.152
Antibacterial agent concomitant with vancomycin therapy^c^, n (%)			
Carbapenems	72 (43.1)	13 (68.4)	0.036
Third generation Cephalosporins	39 (23.4)	4 (21.1)	1.000
Penicillins+β-lactamase inhibitors	29 (17.4)	5 (26.3)	0.351
Fourth generation Cephalosporins	5 (3.0)	0 (0)	1.000
Rifamycin	5 (3.0)	0 (0)	1.000

a The total percentage may >100 due to a patient having multiple infectious sites.

b The total percentage may >100 because a patient received multiple antibacterial drugs before vancomycin therapy.

c The total percentage may >100 because a patient received multiple antibacterial drugs concomitant with vancomycin therapy.

IQR, interquartile range; SD, standard deviation; CRP, C-reactive protein; PCT, procalcitonin; PRISM, Pediatric Risk of Mortality; MSSA, Methicillin susceptible Staphylococcus aureus; MRSA, Methicillin-resistant Staphylococcus aureus; MIC, minimum inhibitory concentration.

### Risk factors for treatment failure

**Figure 2:**
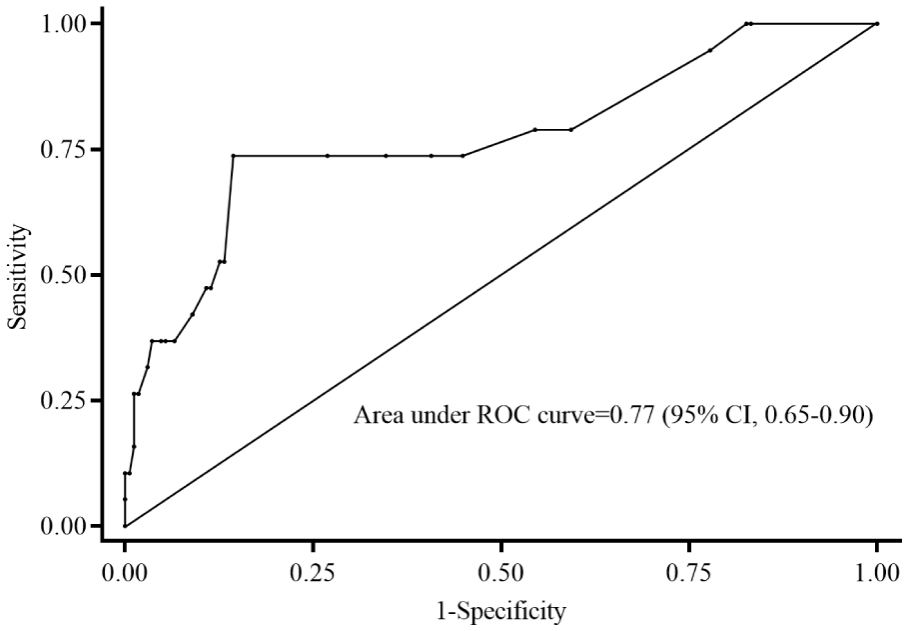
Receiver operating characteristic curve of predictive level of PRISM III scores for treatment failure. The PRISM III score of 10 cut point indicates 73.7% sensitivity and 85.6% specificity on treatment failure. ROC, receiver operating characteristic; CI, confidence interval; PRISM: Pediatric Risk of Mortality.

**Table 2** disclosed that duration of vancomycin therapy, initial and mean vancomycin daily doses, albumin, Gram-negative bacteria co-infections, septic shock, PRISM III scores and carbapenems concomitant with vancomycin therapy might be associated with treatment response. Inter-relationships between PRISM III scores and septic shock may result in confounding of associations between PRISM III scores and treatment failure. Thus, PRISM III scores were finally included in the logistic regression model to avoiding collinearity. The ROC curve (0.77; 95% CI: 0.65-0.90) supported that PRISM III scores could be used to evaluate for treatment failure; PRISM III scores ≥10 could be an appropriate cut point with 73.7% sensitivity and 85.6% specificity in this study (**Figure 2**). Further multivariable logistic analysis revealed that a PRISM III score ≥10 (OR =15.011; 95% CI: 3.937-57.230; P<0.001) was the only independent clinical risk factor for vancomycin treatment failure in these patients (**Table 3**).

**Table 3:** Logistic analysis of risk factors for treatment failure.

Variables	Univariate analysis		Multivariate analysis	
	OR (95% CI)	P Value	OR (95% CI)	P Value
PRISM III scores ≥10	16.683 (5.504-50.566)	<0.001	15.011 (3.937-57.230)	<0.001
Initial vancomycin daily dose (mg/kg/day)	1.067(1.013-1.123)	0.014	1.017 (0.936-1.106)	0.682
Albumin (g/dL)	0.929 (0.867-0.994)	0.033	1.000 (0.924-1.083)	0.997
Mean vancomycin daily dose (mg/kg/day)	1.053 (1.005-1.102)	0.029	1.052 (0.975-1.135)	0.194
Gram negative bacteria co-infections	3.313 (1.189-9.230)	0.022	1.866 (0.519-6.715)	0.339
Carbapenems concomitant with vancomycin therapy	2.859 (1.036-7.886)	0.042	1.110 (0.296-4.159)	0.877
Duration of vancomycin therapy (days)	0.946 (0.876-1.021)	0.155		

OR, odds ratio; CI, confidence interval; PRISM, Pediatric Risk of Mortality.

### The treatment success rate between trough concentrations ≤15 mg/L and >15 mg/L

**Figure 3:**
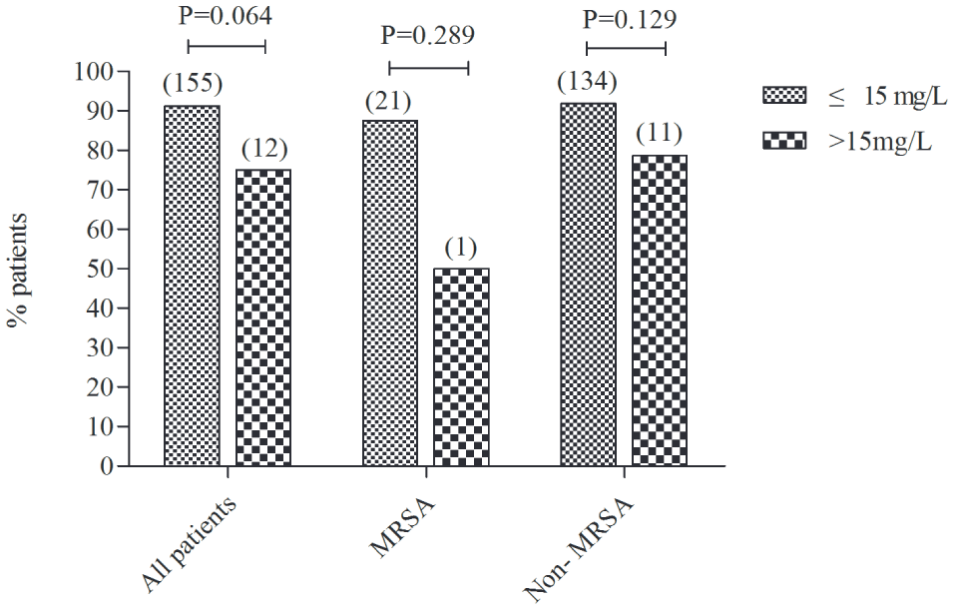
The treatment success rate between trough concentrations ≤15 mg/L and >15 mg/L in all enrolled patients, MRSA infection patients and non-MRSA infection patients. The number in brackets above the columns reflects the total number of children. MRSA, Methicillin-resistant *Staphylococcus aureus*.

The treatment success rate was similar between vancomycin trough concentrations ≤15 mg/L and >15 mg/L in all enrolled patients (91.2% *vs*. 75.0%, P=0.064). We further divided the patients into MRSA infection patients and non-MRSA infection patients and found that the treatment success rate was also no significant differences between trough concentrations ≤15 mg/L and >15 mg/L (87.5% vs. 50.0%, P=0.289; 91.8% vs. 78.6%, P=0.129, respectively) (**Figure 3**).

## Discussion

We found that nearly 90% patients had been treated successfully and there were no vancomycin-related nephrotoxicity adverse effects among all enrolled Gram-positive bacterial sepsis patients. Intriguingly, we found that the treatment success rate was similar between vancomycin trough concentrations ≤15 mg/L and >15 mg/L groups. Furthermore, we demonstrated that a PRISM III score ≥10 was an independent risk factor for treatment failure with the vancomycin dosage of 40-60 mg/kg/d and corresponding trough concentrations in children with Gram-positive bacterial sepsis.

It is widely accepted that appropriate vancomycin dosage is undoubtedly important for children with Gram-positive bacterial sepsis. Our study presented that about 90% patients could be treated successfully with the vancomycin dosage of 40-60 mg/kg/d and corresponding trough concentrations in children with Gram-positive bacterial sepsis, which was similar to the results of the study by Liang et al. (Liang et al., 2018). However, in contrast to our findings, the previously investigators recommended different vancomycin dosages (Rybak et al., 2020; Van Der Heggen et al., 2021). Van Der Heggen et al. suggested that children with severe infections should be treated with 80 mg/kg/d (Van Der Heggen et al., 2021), while the revised US consensus guideline recommended that the initial vancomycin dosage for children with normal renal function and suspected serious MRSA infections should be 60-80 mg/kg/d (Rybak et al., 2020). The reasons for this inconsistency may be attributable to the follows. Firstly, there are different definitions of standards for adjusting vancomycin doses. We combined vancomycin trough concentrations as well as clinical and microbiological therapeutic effects to comprehensively evaluate vancomycin doses, however Van Der Heggen et al. (Van Der Heggen et al., 2021) just used the vancomycin trough concentration of 10-15 mg/L and the revised US consensus guideline (Rybak et al., 2020) based on an AUC target of 400 mg*h/L from adult data to guide the dose adjustment of vancomycin. Secondly, we assessed different bacterial species and infections with different bacterial species, while the revised US consensus guideline focused on MRSA infections or suspected MRSA infections which might correspond to different vancomycin doses recommendation.

It was interesting to note that the treatment success rate was similar between vancomycin trough concentrations ≤15 mg/L and >15 mg/L groups. This observation supports several studies which revealed that vancomycin trough concentrations had no relationship with clinical outcomes (Hirano et al., 2016; Finch et al., 2017; Liang et al., 2018). Currently, guidelines recommend that TDM for children with poor or augmented renal clearance and serious infections and suggest using vancomycin trough concentrations as a surrogate measure for AUC/MIC (Liu et al., 2011; Rybak et al., 2020). Thus, IDSA recommend targeting vancomycin trough concentration of 10-20 mg/L in both adults and pediatrics; 10-15 mg/L for uncomplicated infections and 15-20 mg/L for serious infections (Liu et al., 2011). Nevertheless, the IDSA guidelines do acknowledge that the efficacy of targeting trough concentrations of 15-20 mg/L in children need additional studies due to this recommendation derives from adult studies (Liu et al., 2011). In adults, the trough concentration of 15-20 mg/L had an AUC range from 405 to 792 (Haeseker et al., 2016), while Tang et al. (Tang et al., 2021) recommended an AUC of 240-480 was an optimal exposure target of vancomycin in children. Moreover, some pharmacodynamic studies reported that the trough concentration of 6-10 mg/L was likely sufficient to achieve AUC/MIC ≥400 in children (Frymoyer et al., 2013; Tkachuk et al., 2018). Another recent study also showed that the median vancomycin trough concentration at steady state that related with the AUC/MIC ≥400 and <800 were 11.18, 9.50, 7.91 and 6.55 mg/L in children receiving vancomycin 40, 60, 80 and 100 mg/kg/day, respectively (Issaranggoon Na Ayuthaya et al., 2020). Moreover, researchers have presented that the trough concentration ≥15 mg/L was correlated with nephrotoxicity in children (Le et al., 2015). Therefore, the trough concentration of 15-20 mg/L for children with Gram-positive bacterial sepsis might be reconsidered.

We found that a PRISM III score ≥10 was an independent risk factor for treatment failure with the vancomycin dosage of 40-60 mg/kg/d and corresponding trough concentrations in children with Gram-positive bacterial sepsis. A higher PRISM III score is associated with an increased probability of mortality (Pollack et al., 1996). Yang et al. (Yang et al., 2018) reported that disease severity was the most relevant factor predicting treatment failure in patients with bacteria. Consistent with the observation by Yang et al., findings from our study showed that a PRISM III score ≥10 was a risk factor for treatment failure in children with sepsis. In this study, the failure group patients had a significantly higher proportion of septic shock than that in success group. Severe patients combined with organ dysfunctions and had multi-drug interactions and other therapeutic interventions, which may affect antimicrobial pharmacokinetics (Scaglione and Paraboni, 2008). Hence, caution should be applied when giving a vancomycin dosage of 40-60 mg/kg/day to the sepsis children with PRISM III scores ≥10.

In this study, it was interesting to note that although the failure group patients received a higher vancomycin dosage than that in the success group, they were still treated unsuccessfully. The reasons may be attributable to the follows. One possible explanation might be associated with illness severity. In our research, the failure group patients had significantly higher PRISM III scores than those in the success group. Higher PRISM III scores indicate more severe illness. Thus, some pediatricians might give these severe patients a higher initial vancomycin dosage or adjust to a higher vancomycin dosage, which leaded the initial and mean vancomycin daily doses in failure group were remarkably higher than those in the success group. Another possible explanation might be reaching the target trough concentration of 10-20 mg/L. Truong et al. (Truong et al., 2018) demonstrated that severe sepsis and higher disease severity scores were correlated with higher vancomycin treatment failure rates. Although studies have showed that there was no need to attain trough concentrations of 10-20 mg/L when using AUC-guided vancomycin dosing in children with sepsis (Lanke et al., 2017). Some pediatricians still adjusted the dose of vancomycin based on the target trough concentration of 10-20 mg/L (Sosnin et al., 2019). Therefore, failure group children might be exposed to overuse of vancomycin. Inappropriate use of antibiotics was associated with an elevated risk of antibiotic-related adverse events and healthcare costs (Principi and Esposito, 2019). We have demonstrated PRISM III scores ≥10 might serve as an independent risk factor for vancomycin treatment failure in children with Gram-positive bacterial sepsis. Individualized vancomycin dosages were required in these children. Moreover, the pediatricians should avoid to increase vancomycin dosages blindly in clinical practice when the treatment effect is poor, but should reevaluate the whole treatment strategy. As sepsis comprehensive management not only focuses on antimicrobial treatment, but also pays attention to early identification, source control, appropriate supportive care and hemodynamic optimization (Weiss et al., 2020; Mehta et al., 2022).

We found that failure group patients had a higher proportion of concomitant utilization with carbapenems, but they did not obtain higher treatment success rate. Researches have been reported that vancomycin has many shortcomings, including poor tissue penetration and slow killing time (Tong et al., 2020). Hence, some studies investigated combination therapy using vancomycin with other antibiotics for MRSA infections (Mohammadi-Berenjestanaki et al., 2020; Tong et al., 2020). Mohammadi-Berenjestanaki et al. demonstrated that co-administration of vancomycin and imipenem could effective against MRSA and MSSA infections (Mohammadi-Berenjestanaki et al., 2020). This observation was inconsistent with the results of our study. One possible explanation for this inconsistency could be Mohammadi-Berenjestanaki *et.al*’s study was designed to evaluate the efficacy of combination vancomycin with imipenem only on MRSA and MSSA infections, while our study investigated the efficacy of co-administration of vancomycin and carbapenems on Gram-positive bacterial. Another explanation might be about 74% (14/19) patients in failure group had PRISM III scores ≥10 in our study, which lead to treatment failure. As meropenem therapy was associated with an increased risk of *Clostridioides difficile* infection (Lee et al., 2021). Thus, when there is no evidence of Gram-negative bacterial infections, co-administration of vancomycin and carbapenems should be applied with caution.

There are some limitations of our study. Firstly, the sample size of MRSA-infected children in this study was relatively small, therefore the results of this study should be interpreted with caution when applied to MRSA-infected children. Secondly, we could not estimate the vancomycin nephrotoxicity cutoff value in this population due to no reports of nephrotoxicity adverse effects. Thirdly, although we have tried our best to control the possible bias, it might still exist and potentially skew our results owing to its retrospective design. A prospective randomized multicenter study with larger sample size participants is needed to investigate the vancomycin efficiency and safety with current dosages and corresponding trough concentrations in children with Gram-positive bacterial sepsis.

## Conclusions

Vancomycin dosages of 40-60 mg/kg/d are effective and have no vancomycin-related nephrotoxicity adverse effects occur in children with Gram-positive bacterial sepsis. Vancomycin trough concentrations >15 mg/L are not an essential target for these Gram-positive bacterial sepsis patients. PRISM III scores ≥10 may serve as an independent clinical risk factor for vancomycin treatment failure in these patients.

## Data availability statement

The original contributions presented in the study are included in the article/**Supplementary Material**. Further inquiries can be directed to the corresponding author.

## Ethics statement

The studies involving human participants were reviewed and approved by The Institutional Review Board, Children’s Hospital of Chongqing Medical University. Written informed consent from the participants’ legal guardian/next of kin was not required to participate in this study in accordance with the national legislation and the institutional requirements.

## Author contributions

Conception and design: LP, ZG, and ZL; Methodology: LP, ZG, and GZ; Collection and assembly of data: GZ, XT, and RG; Data analysis and interpretation: LP, QL, and YL, Writing-original draft: LP; Writing- review and editing: ZL. All authors contributed to the article and approved the submitted version.
