# Supplementary material for: Vancomycin efficiency and safety of a dosage of 40–60 mg/kg/d and corresponding trough concentrations in children with Gram-positive bacterial sepsis
Source: Front Cell Infect Microbiol. 2023 Apr 3;13:1117717. doi: 10.3389/fcimb.2023.1117717 (PMC10098341; doi:10.3389/fcimb.2023.1117717)
Supplement: Supplementary file 1 [file Table_1.docx]

Supplementary Material

**Vancomycin efficiency and safety of a dosage of 40-60 mg/kg/d and corresponding trough concentrations in children with Gram-positive bacterial sepsis**

**Lengyue Peng^1,2^, Ziyao Guo^1^, Guangli Zhang^1^, Xiaoyin Tian^1^, Ruixue Gu^1^, Qinyuan Li^1^, Yuanyuan Li^1^, Zhengxiu Luo^1^*******

*** Correspondence:** Zhengxiu Luo: e-mail: [luozhengxiu816@163.com](mailto:luozhengxiu816@163.com)

# Supplementary Data

Supplementary Material should be uploaded separately on submission. Please include any supplementary data, figures and/or tables.

Supplementary material is not typeset so please ensure that all information is clearly presented, the appropriate caption is included in the file and not in the manuscript, and that the style conforms to the rest of the article.

# Supplementary Figures and Tables

For more information on Supplementary Material and for details on the different file types accepted, please see [here](https://www.frontiersin.org/guidelines/author-guidelines#supplementary-material).

Table S1 Liver and kidney function before and after vancomycin treatment of success and failure groups

| Variables | Success group (n=176) | | | Failure group (n=19) | | |
| --- | --- | --- | --- | --- | --- | --- |
|  | Before treatment | After treatment | P Value | Before treatment | After treatment | P Value |
| Liver function |  |  |  |  |  |  |
| Albumin (g/dL), (SD) | 33.6 (7.0) | 36.5 (6.0) | <0.001 | 29.9 (7.2) | 31.6 (6.6) | 0.297 |
| TB (μmol/L), median (IQR) | 5.7 (3.4-9.9) | 4.6 (3.0-7.6) | 0.005 | 7.9 (3.1-13.6) | 5.0 (2.8-9.4) | 0.570 |
| ALT (U/L), median (IQR) | 32.7 (21.7-52.1) | 28.5 (18.0-46.4) | 0.001 | 29.6 (25.1-88.1) | 35.3 (23.9-62.6) | 0.198 |
| AST (U/L), median (IQR) | 31.7 (25.2-51.4) | 33.3 (24.5-49.7) | 0.346 | 45.1 (31.3-102.4) | 42.4 (24.0-72.6) | 0.159 |
| Kidney function |  |  |  |  |  |  |
| Scr (μmol/L), median (IQR) | 25.0 (20.4-33.0) | 25.0 (19.0-30.0) | 0.001 | 21.7 (17.0-30.0) | 18.0 (15.0-31.0) | 0.286 |
| BUN (μmol/L), median (IQR) | 3.3 (2.2-4.4) | 2.6 (1.9-3.7) | <0.001 | 3.4 (1.8-4.8) | 3.0 (1.4-4.8) | 0.212 |

Abbreviations: SD: standard deviation; IQR: interquartile range; TB: total bilirubin; ALT: alanine aminotransferase; AST: aspertate aminotransferase; Scr: serum creatinine; BUN: blood urea nitrogen

Table S2 The data before and after vancomycin concentration adjustment

| Variables | Vancomycin dosage before concentration adjustment, (mg/kg/day), median (IQR) | Vancomycin dosage after concentration adjustment, (mg/kg/day), median (IQR) |
| --- | --- | --- |
| All enrolled patients (n=82) | 40.0 (40.0-57.1) | 58.8 (49.7-60.0) |
| Success group (n=70) | 40.0 (40.0-51.4) | 58.6 (46.9-60.0) |
| Failure group (n=12) | 56.1 (40.0-59.7) | 59.9 (57.3-60.0) |

Abbreviations: IQR: interquartile range

Table S3 The data before and after vancomycin dosage adjustment

| Variables | Vancomycin concentration before dosage adjustment (mg/L), median (IQR) | Vancomycin concentration after dosage adjustment (mg/L), median (IQR) |
| --- | --- | --- |
| All enrolled patients (n=48) | 5.1 (3.9-9.7) | 9.2 (6.2-14.2) |
| Success group (n=40) | 4.9 (3.4-8.9) | 10.2 (6.2-14.7) |
| Failure group (n=8) | 6.6 (4.3-17.0) | 8.1 (6.4-11.9) |

Abbreviations: IQR: interquartile range
